# Targeted UPLC-MS Metabolic Analysis of Human Faeces Reveals Novel Low-Invasive Candidate Markers for Colorectal Cancer

**DOI:** 10.3390/cancers10090300

**Published:** 2018-09-01

**Authors:** Joaquin Cubiella, Marc Clos-Garcia, Cristina Alonso, Ibon Martinez-Arranz, Miriam Perez-Cormenzana, Ziortza Barrenetxea, Jesus Berganza, Isabel Rodríguez-Llopis, Mauro D’Amato, Luis Bujanda, Marta Diaz-Ondina, Juan M. Falcón-Pérez

**Affiliations:** 1 Department of Gastroenterology, Complexo Hospitalario Universitario de Ourense, Instituto de Investigación Biomédica Ourense-Vigo-Pontevedra, 32005 Ourense, Spain; m.ondina@hotmail.com; 2 Exosomes Laboratory, CIC bioGUNE, CIBERehd, Bizkaia Technology Park, Derio, 48160 Bizkaia, Spain; mclos.biodonostia@cicbiogune.es; 3 Department of Gastroenterology, Hospital Donostia/Instituto Biodonostia, Centro de Investigación Biomédica en Red de Enfermedades Hepáticas y Digestivas (CIBERehd), Universidad del País Vasco (UPV/EHU), 20014 San Sebastián, Spain; 4 OWL Metabolomics, Bizkaia Technology Park, Derio, 48160 Bizkaia, Spain; calonso@owlmetabolomics.com (C.A.); imartinez@owlmetabolomics.com (I.M.-A.); mperez@owlmetabolomics.com (M.P.-C.); 5 GAIKER-IK4 Technology Centre, Ed. 202, 48170 Zamudio, Spain; barrenetxea@gaiker.es (Z.B.); berganza@gaiker.es (J.B.); rodriguez@gaiker.es (I.R.-L.); 6 Gastrointestinal Genetics Unit, Biodonostia HRI, 20014 San Sebastián, Spain; mauro.damato.mda@gmail.com; 7 IKERBASQUE, Basque Foundation for Science, 48011 Bilbao, Spain; 8 Metabolomics Platform, CIC bioGUNE, CIBERehd, Bizkaia Technology Park, Derio, 48160 Bizkaia, Spain

**Keywords:** colorectal cancer, metabolomics, faecal samples, biomarkers

## Abstract

Low invasive tests with high sensitivity for colorectal cancer and advanced precancerous lesions will increase adherence rates, and improve clinical outcomes. We have performed an ultra-performance liquid chromatography/time-of-flight mass spectrometry (UPLC-(TOF) MS)-based metabolomics study to identify faecal biomarkers for the detection of patients with advanced neoplasia. A cohort of 80 patients with advanced neoplasia (40 advanced adenomas and 40 colorectal cancers) and 49 healthy subjects were analysed in the study. We evaluated the faecal levels of 105 metabolites including glycerolipids, glycerophospholipids, sterol lipids and sphingolipids. We found 18 metabolites that were significantly altered in patients with advanced neoplasia compared to controls. The combinations of seven metabolites including ChoE(18:1), ChoE(18:2), ChoE(20:4), PE(16:0/18:1), SM(d18:1/23:0), SM(42:3) and TG(54:1), discriminated advanced neoplasia patients from healthy controls. These seven metabolites were employed to construct a predictive model that provides an area under the curve (AUC) median value of 0.821. The inclusion of faecal haemoglobin concentration in the metabolomics signature improved the predictive model to an AUC of 0.885. In silico gene expression analysis of tumour tissue supports our results and puts the differentially expressed metabolites into biological context, showing that glycerolipids and sphingolipids metabolism and GPI-anchor biosynthesis pathways may play a role in tumour progression.

## 1. Introduction

Colorectal cancer (CRC) is the second leading cause of cancer death in developed countries [1]. Although knowledge of the genetic- and diet-associated mechanisms involved in CRC establishment and progression is rapidly increasing [2], still the best prognosis is obtained when malignancy is detected early. CRC screening, which detects both precancerous polyps and CRC, can reduce both colorectal cancer incidence and mortality [3,4,5,6,7]. Through screening, the incidence of colorectal cancer can be reduced by 30% with a mortality reduction of 50% depending on the screening modality and the participation rates [7,8]. These data clearly support the strategy to have efficient and sensitive screening methods. Screening tests available include detecting haemoglobin or DNA mutations/alterations in feces [4,9], radiologic or endoscopic (flexible sigmoidoscopy, colonoscopy, and computed tomographic colonography) methods [10]. Each test has its own advantages, has demonstrated to be cost-effective, and has associated limitations and risks [10]. Although colonoscopy is considered the most accurate test for early detection and prevention of colorectal cancer [11], its applicability is limited due to the secondary effects associated with it (mild and severe), the low adherence in average and familial-risk populations and the limited resources available [12,13]. 

On the other hand, most of CRC are still diagnosed in symptomatic patients, even when CRC screening programs are established [14]. In this regard, symptoms and symptom-based prediction models have a limited accuracy for CRC detection in this population. CRC diagnostic biomarkers, such as faecal haemoglobin, can improve the diagnostic process either alone or within prediction models [15,16,17]. For all those reasons, the development of non-invasive methods to detect CRC either in asymptomatic and symptomatic patients is an area of interest for patients, clinicians and healthcare providers. 

Metabolomics is the omics technology dedicated to the measurement of small molecules (<2000 Da) that are present in a biological system. Major advances and new development of analytical instruments, together with the implementation of bioinformatics tools for robust data analysis allows simultaneous measurement and analysis of a huge number of metabolites from a biological system [18,19,20,21]. In consequence, metabolomics has become one of the main technologies for biomarker identification and for unraveling pathophysiological mechanisms in many diseases, including cancer. The development of ultra-performance liquid chromatography (UPLC) has improved both resolution and sensitivity of metabolomics analysis. It has also allowed the rapid separation of metabolites when compared to conventional LC methods [22,23]. Notably, several metabolomics studies have been performed aiming to identify new CRC biomarkers, as reviewed by Zhang et al. [24]. For diagnostics purpose, several studies exist, although the majority of them have been performed on serum samples [25,26,27,28,29,30,31,32,33], tissue [34,35,36] and urine [37]. To our knowledge, only one study was found that studied metabolomics differences directly in human feces samples, like our project design, using NMR-based metabolomics [38]. Metabolomics study of faeces may be more effective in detecting novel colon cancer makers than other approaches because faeces are in close proximity to the colorectal mucosa and are a product of interactions between dietary components and the microbiota. This latter is affected by and seems to play an important role in the progression of colon cancer [39,40]. Existent literature has identified several metabolites, some being consistently altered in CRC individuals and others being increased in some studies and decreased in other ones [24]. These studies have allowed the identification of several altered metabolic pathways, including carbohydrate and amino acid metabolisms, and lipid-related metabolic pathways. Significantly, most of the studies found differences in metabolites of the tricarboxilic acid (TCA) cycle. Also, importantly, alterations on short-chain fatty acids (SCFAs) levels were found for feces-metabolomics study, which clearly indicates a role for the CRC-specific microbiota composition [38]. Lipid metabolism is an important pathway of cellular energy metabolism and its alteration has been related to CRC development and progression. Alterations on metabolic pathways for the eight distinct pathways of lipid metabolism, including corresponding genes and lipid-specific cell receptors, have been reviewed by Yan et al. 2016 [41].

In this study, we evaluate by UPLC-MS the levels of 105 metabolites in lyophilized faeces from a cohort of 129 samples including patients with advanced adenoma or colon carcinoma and healthy individuals. After applying univariate analysis, we found significant changes between healthy individuals and advanced neoplasia patients in 18 metabolites including sphingomyelins, ceramides, glycerophospholipis and cholesteryl esters. A combined analysis of ChoE(18:1), ChoE(18:2), ChoE(20:4), PE (16:0/18:1), SM(d18:1/23:0), SM(42:3) and TG(54:1) provides an AUC value of 0.821. This work supports the usefulness of metabolomics to develop low invasive diagnostic tools for colon cancer population screenings.

## 2. Results

For the study, we have analysed faecal samples collected from 49 healthy, 40 CRC patients and 40 AD patients (see Materials and Methods for more details). On these samples, we have performed a metabolomics profiling using the UPLC-MS approach as described in Materials and Methods. There is no single method to analyse the entire set of metabolites of a biological sample, mainly due to the wide concentration range of the metabolites joined to their extensive chemical diversity. For this study, we have employed an UPLC-MS method (Supplementary Figure S1) capable of detecting consistently the 105 identified metabolites listed in Supplementary Table S1, that includes fatty acyls, glycerolipids, glycerophospholipids, sterol lipids and sphingolipids.

### 2.1. Multivariate Analysis

**Figure 1:**
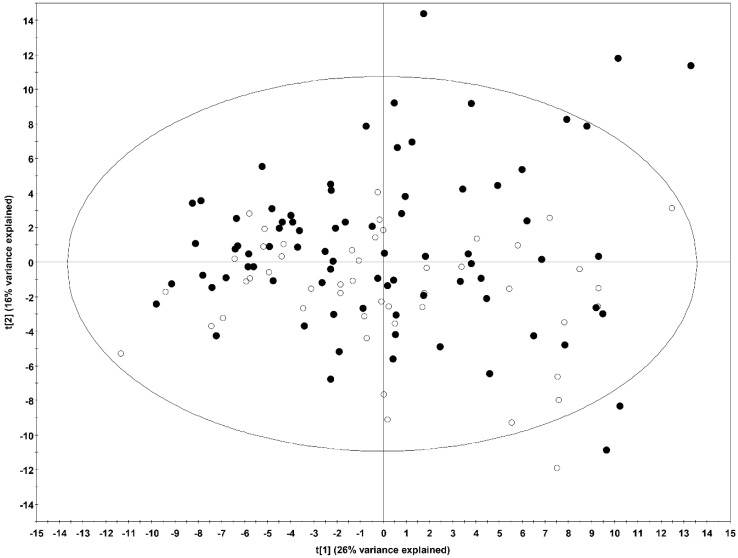
PCA scores plot of healthy individuals and patients with advanced neoplasia. (t[1]: R2X = 0.26 and Q2 = 0.22, t[2]: R2X = 0.16 and Q2 = 0.18): CRC and AD patients (n = 80), filled circles; healthy individuals (n = 49), open circles.

First, we analysed the metabolomic profiling of the 105 metabolites by unsupervised principal component analysis (PCA). We did not find any clustering of samples according to their classification as cases (AD and CRC) and controls (C), as seen on the score plot in Figure 1; neither, did if each group (AD, CRC and C) was compared separately each other (Supplementary Figure S2).

Neither the application of orthogonal (partial least squares) projections to latent structures (OPLS) or multivariate analysis was suitable for obtaining a separation between the groups of samples (data not shown). This lack of discrimination between groups through multivariate analysis highlights the expected high heterogeneity that exists between individuals.

### 2.2. Univariate Analysis

**Figure 2:**
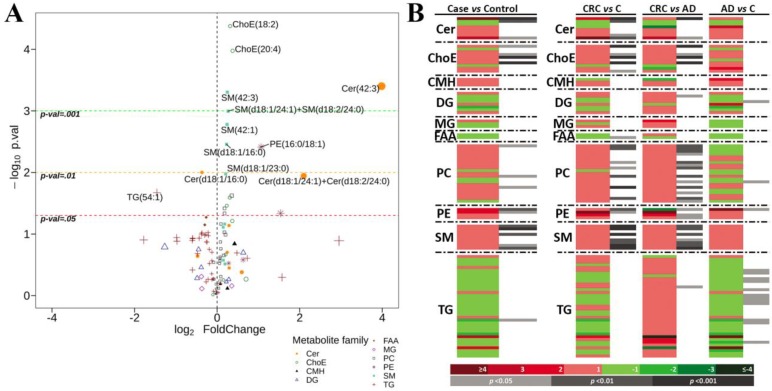
Volcano plot representation of metabolic changes in stools from control, CRC and AD sample groups. [log10 (*p*-value) vs. log2 (fold-change)] for the comparison between healthy individuals and patients with advanced neoplasia (CRC and AD). The shape and colour of the points indicates metabolite family, while the size is determined by the absolute value of the log2 Fold Change (**A**). Heatmap of metabolites altered in stools from control, CRC and AD sample groups (**B**).

As it is complementary to the multivariate analysis, we have applied a univariate approach that has been shown to be an alternative for metabolomics data sets with elevated heterogeneity [26]. The comparison of the 105 metabolites between cases (AD plus CRC) versus control (C) samples, showed significant (adjusted *p*-value < 0.05) difference of the fold change for 18 of them as can be observed in the Volcano plot (Figure 2A). Differences were mostly seen in sphingolipid family (SM and Cer, but not CMH), but also included ChoE, PC, PE and TG metabolites. The most altered metabolite was Cer(42:3), and all metabolites were higher in the case group, except for two of them, Cer(d18:1/16:0) and TG(54:1), which were lower than the control group (Figure 2A). Other highly altered metabolites (log2 fold change < 1) were Cer(d18:1/24:1) + Cer(d18:2/24:0), PE(16:0/18:1), PE(16:0/18:2) and TG(54:1) (Figure 2A).

Paired comparisons of sample groups revealed significant differences for some metabolic classes between CRC and AD, and also between CRC and C individuals (Table 1). Stool samples of patients with CRC had higher levels than AD or C samples of PC and also ChoE and SM metabolite classes. TG family showed the maximum differences when AD was compared to C samples, with alterations in 12 metabolites of the family; it was lower in AD than C. Actually, most of the differences between AD and C groups were found in this metabolite family, with only one metabolite altered for DG, PC and PE families. CMH and MG families did not show any difference in any comparison. 

Ceramides, ChoE, PC and SM metabolite families were consistently increased in cancer samples. Only TG metabolites showed a specific trend for AD samples, being decreased with respect to the control samples, but showing no differences when comparing C versus CRC samples. Only PE family was consistently increased in both CRC and AD samples when compared to C group.

The analysis of the individual metabolites also showed a difference between sample groups (Figure 2B). The heatmaps display the fold change of the 105 metabolites included in the analysis and their significances according to the Student’s t-test for the comparisons performed between CRC and C, CRC and AD and between AD and C groups. In the comparison of case (AD plus CRC) versus C groups, significant metabolites were found mainly in Cer, ChoE, PE and SM families. While the ceramide family included both increased and decreased metabolites; only increased levels of metabolites belonging to ChoE, PC, PE and SM families were found in the case group. 

The comparisons of CRC versus C, and CRC versus AD groups also revealed significant alteration of the levels of metabolites belonging to Cer, ChoE, PE and SM families, but in this case also the abundance of many metabolites belonging to the PC family were significantly altered. Most of the metabolites of these families were elevated in the CRC group in both comparisons. All these changes were not observed when comparing the AD and control groups indicating that those metabolites were mostly altered in the CRC group. Interestingly, a significant down-regulation of metabolites belonging to the TG family was observed mainly in the AD group (Figure 2B).

We also performed ANOVA test to detect significant differences in the metabolic profile between the three groups studies (C vs. AD vs. CRC). As a result, 29 differentially expressed metabolites belonging to Cer, ChoE, PC, PE and SM classes were found to be statistically significant in agreement with the previous paired analysis (Supplementary Table S2). Also, in concordance with the previous analysis, TG altered metabolites showed a specific pattern, being decreased in the AD group.

#### 2.2.1. Predictive Models

**Figure 3:**
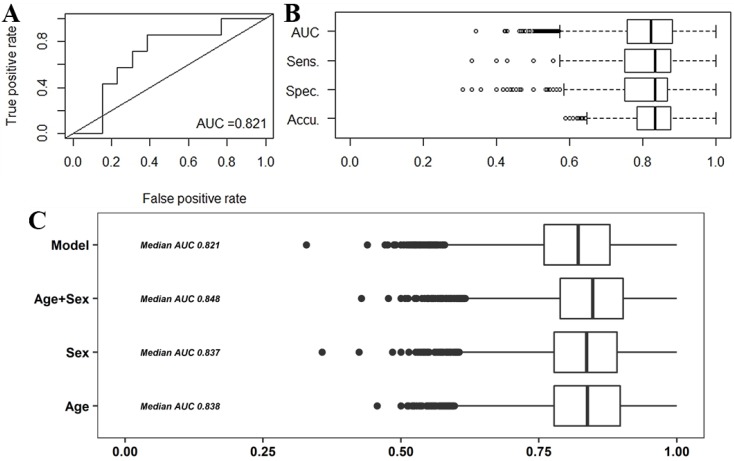
ROC curve of the predictive model constructed with the seven specified metabolites, including the value of the median AUC (**A**). Distribution of the model’s features (AUC, sensitivity, specificity and accuracy) obtained from the 10,000 iterations done (**B**). Distribution of AUC measurements for the combination of our model with age, sex and the age + sex combination (**C**).

In order to construct prediction models for cases (CRC and AD), the cohort was randomly separated in the training set containing 80% of the samples, and the validation set containing the remaining 20% of samples. To avoid possible bias derived from the data separation, we applied a bootstrap method, generating 10,000 different combinations of both training and validation datasets. By applying general linear models to the training set, we were able to find seven metabolites that when combined provide an AUC value of 0.821 (sensitivity 0.833 and specificity 0.800) (Figure 3). The metabolites were ChoE(18:1), ChoE(18:2), ChoE(20:4), PE(16:0/18:1), SM(d18:1/23:0), SM(42:3) and TG(54:1) and the model was:

Y = −5.308 − 1.92 × ChoE(18:2) + 3.087 × ChoE(18:1) − 1.564 × ChoE(20:4) − 1.025 × PE(16:0/18:1) − 0.289 × SM(d18:1/23:0) − 0.678 × SM(42:3) + 0.386 × TG(54:1)

We computed also the potential effects of age and sex upon the performance of our model. We were able to slightly increase the predictive ability of the model when adding the age (AUC = 0.838), sex (AUC = 0.837) and the combination of both (AUC = 0.848) features to the model (Figure 3C). When combining our metabolite model with faecal occult blood (FOB) parameter we were able to increase the AUC value up to 0.885.

#### 2.2.2. Correlation of the Metabolites with Clinical Parameters

A number of clinical parameters were available for the 129 samples analysed in this study including age, gender, FOB test (cut-off 100 ng/mL), carcinoembryonic antigen (CEA) test and COLONPREDICT index. COLONPREDICT is a CRC prediction model that takes into account demographic, symptoms, laboratory and anorectal examination results applicable both in primary and secondary healthcare units [16]. Thus, we evaluated if any of the 105 metabolites analysed in faecal samples correlated with any of the clinical parameters (Supplementary Table S3). There was not strong correlation with age, neither with CEA nor COLONPREDICT or gender, and there were only minor correlations with some clinical data as follows. Several TG metabolites correlated inversely with age data. Also, some metabolites belonging to the DG family correlate with age data, in the same direction as the TG metabolites. COLONPREDICT test showed the highest degree of correlation with metabolites of different families including CMH, PC, ChoE, PE, and SM. Although only slightly, ChoE(18:2) correlated directly with the FOB parameter (Supplementary Table S3).

We also studied how clinical parameters classified samples between the three groups (C, CRC and AD) and between two groups (C and Case) (Supplementary Figure S3). Both ANOVA test for the classification into three groups (Table 2) and Tukey’s HSD test for the classification into two groups (Table 2) showed that COLONPREDICT was the best index to discriminate between samples, followed by FOB. We could see that gender had nearly no differences upon the discrimination between groups, compared to all other clinical parameters. It is important to note that no clinical parameter was able to significantly differentiate between C and AD sample groups.

#### 2.2.3. Gene Expression Analysis of Enzymes Involved in the Metabolism of Altered Metabolites

**Figure 4:**
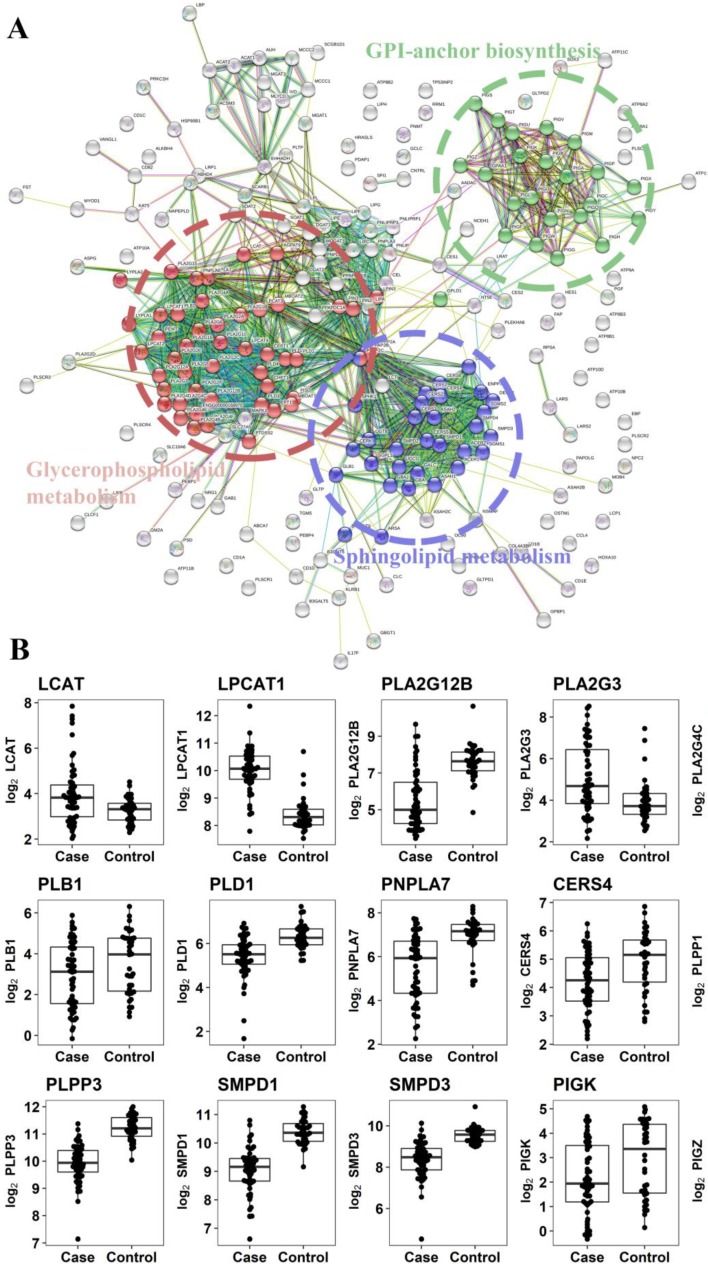
Gene networks of enzymes related with metabolism of stool CRC-altered lipids. Three major pathways could be observed: Sphingolipid and glycerophospholipid metabolisms, and GPI-anchor biosynthesis (**A**). Gene expression in silico analysis of CRC tumoral tissue. The expression of gene-encoding enzymes involved in the metabolism of stool-altered lipids was analysed in publicly available GEO dataset GSE37364 that compared tumoral versus healthy tissue of the same individual. All displayed genes were highly significant (*p*-value < 0.001) except PLPP1 (*p*-value = 0.05) and PIGK (*p*-value = 0.02) (**B**).

Metabolites that were differentially expressed between case and control samples (Figure 2A), and with a KEGG or HMDB code already defined, were employed to identify possible metabolic pathways altered in colorectal cancer. By using the differentially expressed metabolites, we could in-silico identify 211 gene-encoding proteins that mainly clustered in three different metabolic pathways (Figure 4A). The identified pathways were glycerophospholipids metabolism, sphingolipids metabolism and the glycosylphosphatidylinositol (GPI)-anchor biosynthesis pathway suggesting that these pathways could be altered in colorectal cancer (Supplementary Figure S4). We analysed the expression levels of these gene-encoding proteins in the available gene-expression dataset of biopsies of colorectal cancer and normal mucosae of the colon [42]. We have observed that 15 of them showed a significantly different fold change between control and cancer (case) samples (Figure 4B). We have also observed a downregulation of CERS4, SMPD1 and SMPD3 (Figure 4B), which are responsible for the transformation of sphingosines and sphingomyelins to ceramides. We also observed downregulation of genes that encoded enzymes that catalyse the degradation of phosphocholine into choline metabolite, mainly from the phospholipase D (PLD) family: PLB1, PLD1, PNPLA7, PLA2G12B, PLA2G4C (Figure 4B). Furthermore, there was a significant downregulation of the genes PIGK and PIGZ, which encode enzymes involved in GPI-anchor biosynthesis. In addition, an upregulation of the genes LPCAT1 and LCAT (Figure 4B) that encode enzymes involved in the synthesis of phosphatidylcholine and cholesteryl esters, respectively, was also observed. Together, all these alterations on genes involved in lipid metabolism of the tumoral tissue support the lipid changes detected in the faecal samples.

## 3. Discussion

CRC screening with faecal occult blood (FOB) test has demonstrated efficacy in randomized trials. Nonetheless, the low sensitivity for advanced neoplasia of the test suggests the need for more accurate alternative diagnostic tests. In the present study, we have performed an UPLC-based targeted metabolomics analysis of stool to detect candidate endogenous metabolites suitable for the assessment of colon cancer using minimally invasive techniques. Metabolomic study of faeces can be more effective, because faeces are in close proximity to the colorectal mucosa. To date, metabolomics analyses of faecal samples have mostly been restricted to experimental studies in animal and small cross-sectional studies in humans [42,43,44,45,46,47,48,49,50,51,52]. While GC/MS-based metabolic profiling of faecal water has been reported [53,54,55], there exists only limited studies on the profiling and identification of metabolites within the complete faecal material; notably, lyophilized human faeces where its metabotype was confirmed to be more comprehensive than faecal water [47]. Previously, Ponnusamy et al. [56] profiled whole faeces from irritable bowel syndrome using GC/MS and identified several metabolites as candidate biomarkers for the disease. In the current work, a semi-quantitative analysis of 105 metabolites reveals significant differences in the faecal composition of cancer samples in the following lipids: PC(16:0/16:0), PC(32:1), PC(O-16:0/16:0), PE(16:0/18:1), PE(16:0/18:2), SM(d18:1/16:0), SM(d18:1/23:0), SM(d18:2/24:1) + SM(d18:1/24:0), SM(42:1), Cer(d18:1/16:0), Cer(d18:1/24:1) + Cer(d18:2/24:0), Cer(42:1), SM(42:3), ChoE(16:0), ChoE(18:1), ChoE(18:2), ChoE(20:4), TG(54:1). These lipid alterations detected in stools were supported by the gene expression profile observed in tumoral tissues showing deregulation of enzymes involved in glycerophospholipids and the glycosphingolipids metabolisms (Figure 4B). Some of the genes were of special interest as they serve as union nexuses of different metabolic pathways. Thus, PLPP1 and PLPP3 genes encoded lipid phosphate phosphatases (LPPs) with broad substrate specificity that dephosphorylate lipid substrates including phosphatidic acid, lysophosphatidic acid, ceramide 1-phosphate, sphingosine 1-phosphate, and diacylglycerol pyrophosphate [57]. One of their enzymatic reactions is the conversion of phosphatidic acid to diacylglycerol which is a central lipid for glycerophospholipids, triacylglycerols and sphingolipid metabolisms. In consequence, they modulate different signalling pathways and generate building blocks for lipid metabolism-regulating physiological and pathological processes including vascular function and tumor progression [58]. These also indicate that the altered metabolism of the tumour could be detected in stools, and consequently be detected in a non-invasive manner. 

In our study, the most significant lipids altered in stool were cholesteryl esters, particularly ChoE(18:2) and ChoE(20:4) that were increased in CRC samples. This was in agreement with the fact that acetate—a short chain fatty acid—which is the precursor molecule for endogenous cholesterol production, has been reported to be elevated in CRC [59]. In addition, our in silico analysis of the gene expression profile of tumoral tissue reported by Valcz et al. [42] shows increased tumoral levels of the gene encoding the enzyme phosphatidylcholine-sterol acyltransferase responsible for the cholesteryl ester synthesis. Together, the data suggest that the levels of cholesteryl esters in stools can be a suitable non-invasive measurement to detect and follow up colorectal cancer. Based on the cholesteryl esters ChoE(18:1), ChoE(18:2) and ChoE(20:4), and complemented by PE(16:0/18:1), SM(d18:1/23:0), SM(42:3) and TG(54:1), we have built a robust stool metabolomic signature with an AUC value of 0.821 (sensitivity 0.833 and specificity 0.800). In our set of samples, the AUC of the FOB was 0.744, showing that our model of 7-metabolites performed better than the FOB in the detection of CCR. Interestingly, the combination of FOB with our 7-metabolites of our metabolomics model increases the discriminating ability as judged by the AUC value that passed from 0.821 to 0.885. 

It is important to highlight that one of the strengths of our study includes careful processing and preservation of the faecal specimens, and our quantification of within-subject intraclass correlation coefficient (ICC), from which we could estimate statistical power with our cutting-edge faecal metabolomics platform. Our platform has high sensitivity and technical reproducibility, but it has limited ability to detect some volatile and larger molecules. 

Our study’s major limitations are its small size and cross-sectional, hospital-based case–control design. It provided no assessment of temporality and could only detect strong associations with CRC. Also, the fact that this is a targeted metabolomics obviously biases the results towards lipid species, which is also an important limitation. As we mentioned in the introduction section, lipid alterations have been previously associated with CRC development and progression [41]. We considered, therefore, that our panel of metabolites would be sufficient to find potential CRC biomarkers. Also, keeping in mind the diagnostics aim of this study, we decided to use targeted metabolomics because it’s cheaper than an untargeted one, making it a more affordable option. Targeted metabolomics allows an easier interpretation of results and, therefore, an easier translation to clinical practice, which we also considered to be an important point for the diagnostics purpose. As no restriction on diet was provided to the participants in the study, another limitation is the lack of control for potential diet-confounding factors. Nevertheless, we believe this potential diet’s effects to be minimal, as all participants came from two Spanish regions that share the same dietary patterns. We did not specifically control for age, sex, tumour position and staging for this study, which constitutes another important limitation. The decision of not to control for those factors was done taking into account the sample size, not big enough to generate sufficiently big subgroups to obtain statistically robust data. In order to minimize those variables effects, we incorporated the 10,000 iterations through random subsetting of the population for the modelization step, thus generating 10,000 different populations, covering a huge range of different composition trains and test subpopulations that could reduce the potential bias towards some of the mentioned factors. Another strength of our study is the comparison against the FOB test and other clinical parameters. For every one of these comparisons, our model composed by the 7-metabolites performed better than the clinical parameters alone. Also, the integration of gene expression data in the study supports the identification of differentially expressed metabolites and puts them into context, providing some insights on how and why the levels are different between healthy controls and cancer patients. 

## 4. Materials and Methods 

### 4.1. Chemicals

HPLC-MS grade solvents were purchase from Sigma Aldrich (St. Louis, MO, USA). Reference metabolite standard compounds were obtained from Sigma Aldrich, Larodan Fine Chemicals (Malmö, Sweden) and Avanti Polar Lipids (Alabaster, AL, USA).

### 4.2. Clinical Samples and Study Population

The samples were collected during COLONPREDICT study, a multicentre, cross-sectional, blinded study of diagnostic tests aimed to create and validate a CRC prediction index in symptomatic patients based on available biomarkers, clinical and demographical data [16]. The study was approved by the Clinical Research Ethics Committee of Galicia (Code 2011/038). As the samples were collected from the COLONPREDICT study, the population selection characteristics were the same of that study. The cohort consisted of consecutive patients with gastrointestinal symptoms referred for colonoscopy from primary and secondary health care to Complexo Hospitalario Universitario de Ourense, Spain. Exclusion criteria for the COLONPREDICT study were: age under 18, pregnancy, asymptomatic individuals undergoing colonoscopy for CRC screening, patients with previous history of colonic disease, patients requiring hospital admission, patients whose symptoms had ceased within 3 months of evaluation, and patients who declined to participate after reading the informed consent form. Patients self-collected a faecal sample from one bowel movement without specific diet or medication restrictions the week before a colonoscopy was performed at home and delivered to the hospital. The faecal sample was brought to the laboratory in less than 4 hours, split in aliquots and immediately frozen at −80 °C. We selected samples from 40 patients with advanced adenoma-AD- (≥10 mm, villous histology, high-grade dysplasia), 40with CRC and 49 with a normal colonoscopy. The characteristics of the patients differed with respect to age (CRC = 73.1 ± 10.6 years, AD = 68.8 ± 44.6 years, normal = 61.5 ± 14.4 years; *p* < 0.001) and sex (CRC = 60.0% male, AD = 59.1% male, normal = 27.5% male; *p* = 0.004). The CRC were located in the rectum (32.5%), colon distal to splenic flexure (45%) and proximal to splenic flexure (22.5%). The tumour stage at diagnosis was: I (24.2%), II (30.3%), III (30.3%) and IV (15.2%).

### 4.3. Sample Preparation and UPLC®-MS Metabolomics Analysis

A UPLC−time-of-flight (TOF)-MS-based platform was used to analyze chloroform/methanol extracts, including glycerolipids, cholesteryl esters, sphingolipids, primary fatty amides and glycerophospholipids among the identified ion features. The metabolite extraction procedure was as follows. Stools were lyophilized during 3 days by using the instrument Telstar LyoQuest −85. Afterward, 15 milligrams of lyophilized stool samples were mixed with 45 μL sodium chloride (50 mM) and 450 μL chloroform/methanol (30:1) in 1.5 mL microtubes at room temperature. The extraction solvent was spiked with compounds not detected in unspiked human stool samples [SM(d18:1/16:0), PE(17:0/17:0), PC(19:0/19:0), TAG(13:0/13:0/13:0), Cer(d18:1/17:0) and ChoE(12:0)]. After brief vortex mixing, the samples were incubated for 1 hour at −20 °C. After centrifugation at 16,000 × g for 15 min, 35 μL of the lower organic phase was collected and the solvent was removed. The dried extracts were then reconstituted in 1000 μL acetronitrile/isopropanol (1:1), centrifuged (16,000 × g for 5 min), and transferred to vials for UPLC®-MS analysis on an Acquity-Xevo G2 QTof system (Waters Corp., Milford, MA, USA). Samples were randomly divided into three batches, which contained a maximum of 78 samples. Chromatographic method and mass spectrometric detection conditions were described by Barr et al. [60]. Of the different platforms described, the one corresponding to ours was Platform 3.

### 4.4. Data Pre-Processing

Data pre-processing was processed using the TargetLynx application manager for MassLynx 4.1 (Waters Corp). A total of 105 UPLC-MS features were analysed, all of them identified prior to the analysis. Peak detection and noise reduction were performed as previously described [61,62]. Intra- and inter-batch normalization process was based on multiple internal standards and the pool calibration samples approach described by Martinez-Arranz et al. [62].

### 4.5. Data Analysis

The biomarker assessment in this study was organized in sequential and consecutive phases for discovery and biological validation. Firstly, 133 metabolites including glycerolipids, glycerophospholipids, sterol lipids and sphingolipids were selected as candidate biomarkers for initial analysis faeces samples from advanced neoplasia cases, colorectal cancer and cancer-free controls (Discovery Phase). Secondly, the potential clinical use of the most promising validated candidates was tested in faeces samples from colon cancer cases, a small set of adenomas, and cancer-free controls. Reported STARD guidelines have been the basis for defining our protocol.

Metabolites with less than 70% of the values present were removed from the analysis (remaining 105 metabolites into the analysis). Remaining missing values were imputed metabolite by metabolite, taking the minimal value for the metabolite and dividing it by 10. Data was then normalized with the log10 transformation. Univariate statistical analyses were also performed calculating group percentage changes and the analysis of variance (ANOVA) for the comparison among the different groups: CRC, AD and control (C). Student’s t-test *p*-values were calculated for the comparison between cases (AD and CRC) and C groups, as well as for the comparisons CRC and C, CRC and AD and between AD and C groups. Multivariate analyses were also performed, including both Principal Component Analysis (PCA) and Partial Least Squares Discriminant Analysis one (PLS-DA). ANOVA tests and Tukey’s HSD tests were also calculated for several clinical parameters (FOB, sex, age, CEA and COLONPREDICT test) to determine its effectiveness to classify our samples into categories (CRC, AD and C or Case-Control). All *p*-values were adjusted with Bonferroni methodology unless otherwise stated.

A logistic regression (LR) was performed to identify a predictive signature capable of distinguishing between cases and control groups. LR is a commonly used technique for data classification. We first analysed the correlations between metabolites, establishing a cut-off at ρ 0.75. For each pair of correlated metabolites, we removed the one that separated the worst out of the two groups. A forward stepwise method was selected as variable selection approach, where the analysis started with an empty model and variables were added one at a time as long as these additions are worthy, by measuring the Area Under the Curve (AUC) value. This process finished when no more variables could be added. All samples were randomly divided into estimation (80% of all subjects; n = 101) and validation (20% of all subjects; n = 26) groups, both cohorts having an equal proportional representation of individuals belonging to cases and control groups. Ten-thousand iterations of both subsetting into estimation and validation groups and model constructing were generated, to avoid population-based biases. Receiver operating characteristic (ROC) curve analysis was used to assess its discriminatory power. Overall diagnostic accuracy for a given two-class comparison was given by the area under the ROC curve (AUC) with its associated standard error. Sensitivity, specificity and accuracy values were calculated.

All calculations were performed using statistical software package R v.3.1.1 (R Development Core Team, 2011; http://cran.r-project.org) with caret, caTools and receiver operating characteristic R (ROCR) packages to produce ROC curves and AUC estimate; MASS package was used to generate the LR. Additionally, SIMCA-P+ 12.0.1 (Umetrics AB, Umeå, Sweden) was used for PCA and PLS-DA multivariate data analysis.

Retrieval of genes and enzymes related with differentially expressed metabolites found in the study was done with custom Python scripts, which takes advantage of the published Python packages Biopython [63] and bioservices [64], which were used to access both HMDB and KEGG databases. These custom scripts retrieve information on the metabolite entries on both HMDB and KEGG databases regarding the enzymes involved in the metabolism of cited metabolites, as in which pathways are they present. We identified gene-encoding proteins involved in the metabolism of the seven metabolites of the predictive model, and we uploaded those genes to the STRING database [65], in order to identify the interaction between them, any potential clusterization and possible affected metabolic pathways. Genetic expression was obtained from publicly available GEO databaset GSE37364 [42]. The datasets were uploaded to R and the expression of selected genes was plotted into boxplots. Mapping of both metabolites and genes into metabolic pathways was done with pathview package [66] and custom R scripts.

## 5. Conclusions

This study highlights the power of UPLC-MS-based metabolomics approach in the discovery of novel non-invasive markers for colorectal cancer. With this study, we identified alterations in two main metabolic pathways, the glycerophospholipids and glycosphingolipids metabolisms. We found 18 metabolites differentially expressed between case samples (CRC + AD) and healthy controls, being mainly increased in case ones. We also showed how a discrimination model based only on metabolite species was able to differentiate between case (CRC+AD) samples and healthy ones and is better than those used nowadays, based in several clinical parameters like FOB, CEA, etc. The model generated included these metabolites: ChoE(18:1), ChoE(18:2), ChoE(20:4), PE (16:0/18:1), SM(d18:1/23:0), SM(42:3) and TG(54:1). Finally, we showed how the integration of different omics technologies might be useful for supporting findings of one of them and to gain insights on how to explain the results obtained.

## Figures and Tables

**Table 1:** Alteration in metabolic classes. Number of metabolites per metabolic classes differentially expressed in cases vs. control (C), CRC vs. AD, and CRC vs. control. Arrows indicate if metabolites are higher (↑), or lower (↓) in the Case, CRC or AD, depending on the comparison. In parentheses, the number of metabolites analyzed for each family is indicated.

	Case vs. Control	C vs. CRC	C vs. AD	AD vs. CRC
**Cer _(8)_**	2↑ 1↓	3↑ 1↓	0	2↑
**ChoE _(10)_**	4↑	5↑	0	4↑
**CMH _(3)_**	0	0	0	0
**DG _(8)_**	0	0	1↓	1↑
**MG _(3)_**	0	0	0	0
**FAA _(2)_**	0	1↓	0	0
**PC _(21)_**	3↑	7↑	1↓	13↑
**PE _(4)_**	2↑	2↑	1↑	3↑
**SM _(9)_**	5↑	7↑	0	7↑
**TG _(37)_**	1↓	0	12↓	1↑

**Table 2:** Differences between sample classification of several clinical parameters, either for the groups comparison (C, AD and CRC) and for the pairwise comparison (Control vs. Case). ANOVA test has been used for the study of differences between the three groups classification (C, AD and CRC) and Tukey’s HSD test was used to analyse pairwise classifications (C vs. AD, C vs. CRC and AD vs. CRC). Tukey’s HSD column depicts those pairwise combinations (of the three tested combinations) that showed to be significantly different. Avg. stands for average.

**C, AD and CRC**	**Avg_C_**	**Avg_AD_**	**Avg_CRC_**	***p*-Value**	**Tukey’s HSD**
**Gender**	35.4% men	56.4% men	60% men	0.042	*NA*
**Age**	62.52	68.64	73.50	0.0003	CRC vs. C
FOB *	0	49	873	1.6 × 10^−9^	CRC vs. C CRC vs. AD
**CEA**	1.90	1.72	14.85	0.00546	CRC vs. C CRC vs. AD
**COLONPREDICT**	0.048	0.104	0.470	<2 × 10^−16^	CRC vs. C CRC vs. AD
**Control vs. Case**	**Avg_CONTROL_**	**Avg_CASE_**	***p*-Value**		
**Gender**	35.4% men	58.3% men	0.013		
**Age**	62.52	71.10	0.00083		
FOB *	0	336	7.09 × 10^−8^		
**CEA**	1.900	8.367	0.0036		
**COLONPREDICT**	0.0477	0.289	1.231 × 10^−10^		

* For FOB index, median values are given instead of mean, due to the non-normal distribution of the measurements.

## Supplementary Materials

The following are available online at http://www.mdpi.com/2072-6694/10/9/300/s1, Supplementary Figure S1, Workflow of the UPLC-MS-based targeted metabolomic profiling. Supplementary Figure S2, Multivariate analysis of paired group. (A) CRC vs. AD: R2X = 0.29 and Q2 = 0.24 t[2]: R2X = 0.19 and Q2 = 0.22). Black CRC, grey AD. (B) CRC vs. control: R2X = 0.30 and Q2 = 0.25, t[2]: R2X = 0.19 and Q2 = 0.24). Black CRC, white healthy. (C) AD vs. control: R2X = 0.28 and Q2 = 0.24, t[2]: R2X = 0.15 and Q2 = 0.15. Grey AD, white healthy. Supplementary Figure S3, Boxplot representation of the clinical parameters distribution on the distinct groups of samples (C, AD and CRC). Supplementary Figure S4, Mapping of altered genes and metabolites into the three metabolic pathways identified: sphingolipid metabolism (A), glycerophospholipid metabolism (B) and glycosylphosphatidylinositol (GPI)-anchor biosynthesis (C). Genes detected are coloured in a range green-red, depending on the Fold Change and metabolites in a range blue-yellow. Supplementary Table S1, List of the 105 metabolites analysed in the study. Supplementary Table S2, Metabolites differentially expressed between control, AD and CRC groups (ANOVA test). Supplementary Table S3, Clinical correlations between metabolites included in the study and the following parameters: FOB, Sex, Age, COLONPREDICT and CEA. Supplementary Table S4, Number of missing values obtained for each metabolite.

## Abbreviations

AUC	Area Under the Curve
CEA	Carcinoembryonic Antigen
Cer	Ceramides
ChoE	Cholesteryl esters
CMH	Monohexosylceramides
DAG	Diacylglycerides
DAPC	Diacylglycerophosphocholines
FAA	Fatty acid amides (Primary Fatty Amides)
FOB	Faecal Occult Blood
MAG	Monoacylglycerides
MEMAPC	1-ether, 2-acylglycerophosphocholines
PC	Phosphatidylcholines
PCA	Principal Component Analysis
PE	Phosphatidylethanolamines
PI	Phosphatidylinositols
PLS-DA	Partial Least Square Discriminant Analysis
SM	Sphingomyelins
TAG	Triacylglycerides
UPLC®-MS	Ultra performance liquid chromatography-mass spectrometry

## References

[1] Ferlay J., Soerjomataram I., Dikshit R., Eser S., Mathers C., Rebelo M., Parkin D.M., Forman D., Bray F. (2015). Cancer incidence and mortality worldwide: Sources, methods and major patterns in GLOBOCAN 2012. Int. J. Cancer.

[2] Vogelstein B., Papadopoulos N., Velculescu V.E., Zhou S., Diaz L.A., Kinzler K.W. (2013). Cancer Genome Landscapes. Science.

[3] Zauber A.G., Winawer S.J., O’Brien M.J., Lansdrop-Vogelaar I., van Ballegooijen M., Hankey B.F., Shi W., Bond J.H., Schapiro M., Panish J.F. (2012). Colonoscopic Polypectomy and Long-Term Prevention of Colorectal-Cancer Deaths. N. Engl. J. Med..

[4] Quintero E., Castells A., Bujanda L., Cubiella J., Salas D., Lanas Á., Andreu M., Hernández C., Jover R., Montalvo I. (2015). Colonoscopy versus Fecal Immunochemical Testing in Colorectal-Cancer Screening. N. Engl. J. Med..

[5] Lindholm E., Brevinge H., Haglind E. (2008). Survival benefit in a randomized clinical trial of faecal occult blood screening for colorectal cancer. Br. J. Surg..

[6] Faivre J., Dancourt V., Lejeune C., Tazi M.A., Lamour J., Gerard D., Dassonville F., Bonithon-Kopp C. (2004). Reduction in colorectal cancer mortality by fecal occult blood screening in a French controlled study. Gastroenterology.

[7] Atkin W.S., Edwards R., Kralj-Hans I., Wooldrage K., Hart A.R., Northover J.M., Parkin D.M., Wardle J., Duffy S.W., Cuzick J. (2010). Once-only flexible sigmoidoscopy screening in prevention of colorectal cancer: A multicentre randomised controlled trial. Lancet.

[8] Segnan N., Senore C., Andreoni B., Arrigoni A., Bisanti L., Cardelli A., Castiglione G., Crosta C., DiPlacido R., Ferrari A. (2005). Randomized trial of different screening strategies for colorectal cancer: Patient response and detection rates. J. Natl. Cancer Inst..

[9] Imperiale T.F., Ransohoff D.F., Itzkowitz S.H., Levin T.R., Lavin P., Lidgard G.P., Ahlquist D.A., Berger B.M. (2014). Multitarget Stool DNA Testing for Colorectal-Cancer Screening. N. Engl. J. Med..

[10] Levin B., Lieberman D.A., McFarland B., Smith R.A., Brooks D., Andrews K.S., Dash C., Giardiello F.M., Glick S., Levin T.R. (2008). Screening and Surveillance for the Early Detection of Colorectal Cancer and Adenomatous Polyps, 2008: A Joint Guideline from the American Cancer Society, the US Multi-Society Task Force on Colorectal Cancer, and the American College of Radiology. CA Cancer J. Clin..

[11] Regula J., Rupinski M., Kraszewska E., Polkowski M., Pachlewski J., Orlowska J., Nowacki M.P., Butruk E. (2006). Colonoscopy Screening for Detection of Advanced Neoplasia. N. Engl. J. Med..

[12] Bujanda L., Sarasqueta C., Zubiaurre L., Cosme A., Muñoz C., Sánchez A., Martín C., Tito L., Piñol V., Castells A. (2007). Low adherence to colonoscopy in the screening of first-degree relatives of patients with colorectal cancer. Gut.

[13] Puente Gutiérrez J.J., Marín Moreno M.A., Domínguez Jiménez J.L., Bernal Blanco E., Díaz Iglesias J.M. (2011). Effectiveness of a colonoscopic screening programme in first-degree relatives of patients with colorectal cancer. Color. Dis..

[14] Mansouri D., McMillan D.C., Crearie C., Morrison D.S., Crighton E.M., Horgan P.G. (2015). Temporal trends in mode, site and stage of presentation with the introduction of colorectal cancer screening: A decade of experience from the West of Scotland. Br. J. Cancer.

[15] Cubiella J., Digby J., Rodríguez-Alonso L., Vega P., Salve M., Díaz-Ondina M., Strachan J.A., Mowat C., McDonald P.J., Carey F.A. (2017). The fecal hemoglobin concentration, age and sex test score: Development and external validation of a simple prediction tool for colorectal cancer detection in symptomatic patients. Int. J. Cancer.

[16] Cubiella J., Vega P., Salve M., Díaz-Ondina M., Alves M.T., Quintero E., Álvarez-Sánchez V., Fernández-Bañares F., Boadas J., Campo R. (2016). COLONPREDICT study investigators Development and external validation of a faecal immunochemical test-based prediction model for colorectal cancer detection in symptomatic patients. BMC Med..

[17] Westwood M., Lang S., Armstrong N., van Turenhout S., Cubiella J., Stirk L., Ramos I.C., Luyendijk M., Zaim R., Kleijnen J. (2017). Faecal immunochemical tests (FIT) can help to rule out colorectal cancer in patients presenting in primary care with lower abdominal symptoms: A systematic review conducted to inform new NICE DG30 diagnostic guidance. BMC Med..

[18] Chen C., Gonzalez F.J., Idle J.R. (2007). LC-MS-based metabolomics in drug metabolism. Drug Metab. Rev..

[19] Clarke C.J., Haselden J.N. (2008). Metabolic Profiling as a Tool for Understanding Mechanisms of Toxicity. Toxicol. Pathol..

[20] Fernie A.R., Trethewey R.N., Krotzky A.J., Willmitzer L. (2004). Metabolite profiling: from diganostics to systems biology. Nat. Rev. Mol. Cell Biol..

[21] Nicholson J.K., Wilson I.D. (2003). Understanding “global” systems biology: Metabonomics and the continuum of metabolism. Nat. Rev. Drug Discov..

[22] Nordström A., O’Maille G., Qin C., Siuzdak G. (2006). Non-linear Data Alignment for UPLC-MS and HPLC-MS based Metabolomics: Application to Endogenous and Exogenous Metabolites in Human Serum. Anal Chem.

[23] Nováková L., Solichová D., Solich P. (2006). Advantages of ultra performance liquid chromatography over high-performance liquid chromatography: Comparison of different analytical approaches during analysis of diclofenac gel. J. Sep. Sci..

[24] Zhang F., Zhang Y., Zhao W., Deng K., Wang Z., Yang C., Ma L., Openkova M.S., Hou Y., Li K. (2017). Metabolomics for biomarker discovery in the diagnosis, prognosis, survival and recurrence of colorectal cancer: a systematic review. Oncotarget.

[25] Cross A.J., Moore S.C., Boca S., Huang W.-Y., Xiong X., Stolzenberg-Solomon R., Sinha R., Sampson J.N. (2014). A prospective study of serum metabolites and colorectal cancer risk. Cancer.

[26] Ikeda A., Nishiumi S., Shinohara M., Yoshie T., Hatano N., Okuno T., Bamba T., Fukusaki E., Takenawa T., Azuma T. (2012). Serum metabolomics as a novel diagnostic approach for gastrointestinal cancer. Biomed. Chromatogr..

[27] Leichtle A.B., Nuoffer J.M., Ceglarek U., Kase J., Conrad T., Witzigmann H., Thiery J., Fiedler G.M. (2012). Serum amino acid profiles and their alterations in colorectal cancer. Metabolomics.

[28] Li F., Qin X., Chen H., Qiu L., Guo Y., Liu H., Chen G., Song G., Wang X., Li F. (2013). Lipid profiling for early diagnosis and progression of colorectal cancer using direct-infusion electrospray ionization Fourier transform ion cyclotron resonance mass spectrometry. Rapid Commun. Mass Spectrom..

[29] Nishiumi S., Kobayashi T., Ikeda A., Yoshie T., Kibi M., Izumi Y., Okuno T., Hayashi N., Kawano S., Takenawa T. (2012). A novel serum metabolomics-based diagnostic approach for colorectal cancer. PLoS ONE.

[30] Ma Y., Zhang P., Wang F., Liu W., Yang J., Qin H. (2012). An integrated proteomics and metabolomics approach for defining oncofetal biomarkers in the colorectal cancer. Ann. Surg..

[31] Ritchie S.A., Ahiahonu P.W.K., Jayasinghe D., Heath D., Liu J., Lu Y., Jin W., Kavianpour A., Yamazaki Y., Khan A.M. (2010). Reduced levels of hydroxylated, polyunsaturated ultra long-chain fatty acids in the serum of colorectal cancer patients: implications for early screening and detection. BMC Med..

[32] Tan B., Qiu Y., Zou X., Chen T., Xie G., Cheng Y., Dong T., Zhao L., Feng B., Hu X. (2013). Metabonomics Identi fi es Serum Metabolite Markers of Colorectal Cancer. J. Proteome Res..

[33] Zhu J., Djukovic D., Deng L., Gu H., Himmati F., Chiorean E.G., Raftery D. (2014). Colorectal cancer detection using targeted serum metabolic profiling. J. Proteome Res..

[34] Manna S.K., Tanaka N., Krausz K.W., Haznadar M., Xue X., Matsubara T., Bowman E.D., Fearon E.R., Harris C.C., Shah Y.M. (2014). Biomarkers of coordinate metabolic reprogramming in colorectal tumors in mice and humans. Gastroenterology.

[35] Mirnezami R., Jiménez B., Li J.V., Kinross J.M., Veselkov K., Goldin R.D., Holmes E., Nicholson J.K., Darzi A. (2014). Rapid diagnosis and staging of colorectal cancer via high-resolution magic angle spinning nuclear magnetic resonance (HR-MAS NMR) spectroscopy of intact tissue biopsies. Ann. Surg..

[36] Wang H., Wang L., Zhang H., Deng P., Chen J., Zhou B., Hu J., Zou J., Lu W., Xiang P. (2013). ^1^H NMR-based metabolic profiling of human rectal cancer tissue. Mol. Cancer.

[37] Silva C.L., Passos M., Cmara J.S. (2011). Investigation of urinary volatile organic metabolites as potential cancer biomarkers by solid-phase microextraction in combination with gas chromatography-mass spectrometry. Br. J. Cancer.

[38] Lin Y., Ma C., Liu C., Wang Z., Yang J., Liu X., Shen Z., Wu R. (2016). NMR-based fecal metabolomics fingerprinting as predictors of earlier diagnosis in patients with colorectal cancer. Oncotarget.

[39] Irrazábal T., Belcheva A., Girardin S.E., Martin A., Philpott D.J. (2014). The multifaceted role of the intestinal microbiota in colon cancer. Mol. Cell.

[40] Gao Z., Guo B., Gao R., Zhu Q., Qin H. (2015). Microbiota disbiosis is associated with colorectal cancer. Front. Microbiol..

[41] Yan G., Li L., Zhu B., Li Y. (2016). Lipidome in colorectal cancer. Oncotarget.

[42] Valcz G., Patai Á.V., Kalmár A., Péterfia B., Furi I., Wichmann B., Muzes G., Sipos F., Krenács T., Mihály E. (2014). Myofibroblast-derived SFRP1 as potential inhibitor of colorectal carcinoma field effect. PLoS ONE.

[43] Chow J., Panasevich M.R., Alexander D., Vester Boler B.M., Rossoni Serao M.C., Faber T.A., Bauer L.L., Fahey G.C. (2014). Fecal metabolomics of healthy breast-fed versus formula-fed infants before and during in vitro batch culture fermentation. J. Proteome Res..

[44] Zheng X., Xie G., Zhao A., Zhao L., Yao C., Chiu N.H.L., Zhou Z., Bao Y., Jia W., Nicholson J.K. (2011). The Footprints of Gut Microbial-Mammalian Co-Metabolism. J. Proteome Res..

[45] Jump R.L.P., Polinkovsky A., Hurless K., Sitzlar B., Eckart K., Tomas M., Deshpande A., Nerandzic M.M., Donskey C.J. (2014). Metabolomics analysis identifies intestinal microbiota-derived biomarkers of colonization resistance in clindamycin-treated mice. PLoS ONE.

[46] Martin F.J., Sprenger N., Montoliu I., Rezzi S., Kochhar S., Nicholson J.K. (2010). Dietary Modulation of Gut Functional Ecology Studied by Fecal Metabonomics Francois-Pierre. J. Proteome Res..

[47] Phua L.C., Koh P.K., Cheah P.Y., Ho H.K., Chan E.C.Y. (2013). Global gas chromatography/time-of-flight mass spectrometry (GC/TOFMS)-based metabonomic profiling of lyophilized human feces. J. Chromatogr. B Anal. Technol. Biomed. Life Sci..

[48] Saric J., Wang Y., Li J., Coen M., Utzinger J., Marchesi J.R., Keiser J., Veselkov K., Lindon J.C., Nicholson J.K. (2008). Species variation in the fecal metabolome gives insight into differential gastrointestinal function. J. Proteome Res..

[49] Stella C., Beckwith-Hall B., Cloarec O., Holmes E., Lindon J.C., Powell J., Van Der Ouderaa F., Bingham S., Cross A.J., Nicholson J.K. (2006). Susceptibility of human metabolic phenotypes to dietary modulation. J. Proteome Res..

[50] Weir T.L., Manter D.K., Sheflin A.M., Barnett B.A., Heuberger A.L., Ryan E.P. (2013). Stool Microbiome and Metabolome Differences between Colorectal Cancer Patients and Healthy Adults. PLoS ONE.

[51] Xu W., Chen D., Wang N., Zhang T., Zhou R., Huan T., Lu Y., Su X., Xie Q., Li L. (2017). Development of High-Performance Chemical Isotope Labeling LC-MS for Profiling the Human Fecal Metabolome. Anal. Chem..

[52] Zhao Y., Wu J., Li J.V., Zhou N., Tang H., Wang Y. (2013). Gut Microbiota Composition Modifies Fecal Metabolic Profiles in Mice. J. Proteome Res..

[53] Gao X., Pujos-Guillot E., Martin J.F., Galan P., Juste C., Jia W., Sebedio J.L. (2009). Metabolite analysis of human fecal water by gas chromatography/mass spectrometry with ethyl chloroformate derivatization. Anal. Biochem..

[54] Gao X., Pujos-Guillot E., Sébédio J.L. (2010). Development of a quantitative metabolomic approach to study clinical human fecal water metabolome based on trimethylsilylation derivatization and GC/MS analysis. Anal. Chem..

[55] Poroyko V., Morowitz M., Bell T., Ulanov A., Wang M., Donovan S., Bao N., Gu S., Hong L., Alverdy J.C. (2011). Diet creates metabolic niches in the “inmature gut” that shape microbial communities. Nutr. Hosp..

[56] Ponnusamy K., Choi J.N., Kim J., Lee S.Y., Lee C.H. (2011). Microbial community and metabolomic comparison of irritable bowel syndrome faeces. J. Med. Microbiol..

[57] Sciorra V.A., Morris A.J. (2002). Roles for lipid phosphate phosphatases in regulation of cellular signaling. Biochim. Biophys. Acta-Mol. Cell Biol. Lipids.

[58] Tang X., Benesch M.G.K., Brindley D.N. (2015). Lipid phosphate phosphatases and their roles in mammalian physiology and pathology. J. Lipid Res..

[59] Weaver G.A., Krause J.A., Miller T.L., Wolin M.J. (1988). Short chain fatty acid distribution of enema samples from a sigmoidoscopy population:an association of high acetate and low butyrate ratios with adenomatous polyps and colon cancer. Gut.

[60] Barr J., Caballería J., Martínez-Arranz I., Domínguez-Díez A., Alonso C., Muntané J., Pérez-Cormenzana M., García-Monzón C., Mayo R., Martín-Duce A. (2012). Obesity-dependent metabolic signatures associated with nonalcoholic fatty liver disease progression. J. Proteome Res..

[61] Saccenti E., Hoefsloot H.C.J., Smilde A.K., Westerhuis J.A., Hendriks M.M.W.B. (2014). Reflections on univariate and multivariate analysis of metabolomics data. Metabolomics.

[62] Martínez-Arranz I., Mayo R., Pérez-Cormenzana M., Mincholé I., Salazar L., Alonso C., Mato J.M. (2015). Enhancing metabolomics research through data mining. J. Proteomics.

[63] Cock P.J.A., Antao T., Chang J.T., Chapman B.A., Cox C.J., Dalke A., Friedberg I., Hamelryck T., Kauff F., Wilczynski B. (2009). Biopython: Freely available Python tools for computational molecular biology and bioinformatics. Bioinformatics.

[64] Cokelaer T., Pultz D., Harder L.M., Serra-Musach J., Saez-Rodriguez J., Valencia A. (2013). BioServices: A common Python package to access biological Web Services programmatically. Bioinformatics.

[65] Szklarczyk D., Morris J.H., Cook H., Kuhn M., Wyder S., Simonovic M., Santos A., Doncheva N.T., Roth A., Bork P. (2017). The STRING database in 2017: quality-controlled protein-protein association networks, made broadly accessible. Nucleic Acids Res..

[66] Luo W., Brouwer C. (2013). Pathview: An R/Bioconductor package for pathway-based data integration and visualization. Bioinformatics.

