# Supplementary material for: Targeted UPLC-MS Metabolic Analysis of Human Faeces Reveals Novel Low-Invasive Candidate Markers for Colorectal Cancer
Source: Cancers (Basel). 2018 Sep 1;10(9):300. doi: 10.3390/cancers10090300 (PMC6162413; doi:10.3390/cancers10090300)
Supplement: Supplementary file 1 [file cancers-10-00300-s001.zip › cancers-334590-supplementary Figure S1-S4 and Table S2-final.pdf]

# Targeted UPLC-MS Metabolic Analysis of Human Faeces Reveals Novel Low-Invasive Candidate Markers for Colorectal Cancer

**Joaquin Cubiella, Marc Clos-Garcia, Cristina Alonso, Ibon Martinez-Arranz, Miriam Perez-Cormenzana, Ziortza Barrenetxea, Jesus Berganza, Isabel Rodríguez-Llopis, Mauro D´Amato, Luis Bujanda, Marta Diaz-Ondina and Juan M. Falcón-Pérez**

## Supplementary Materials

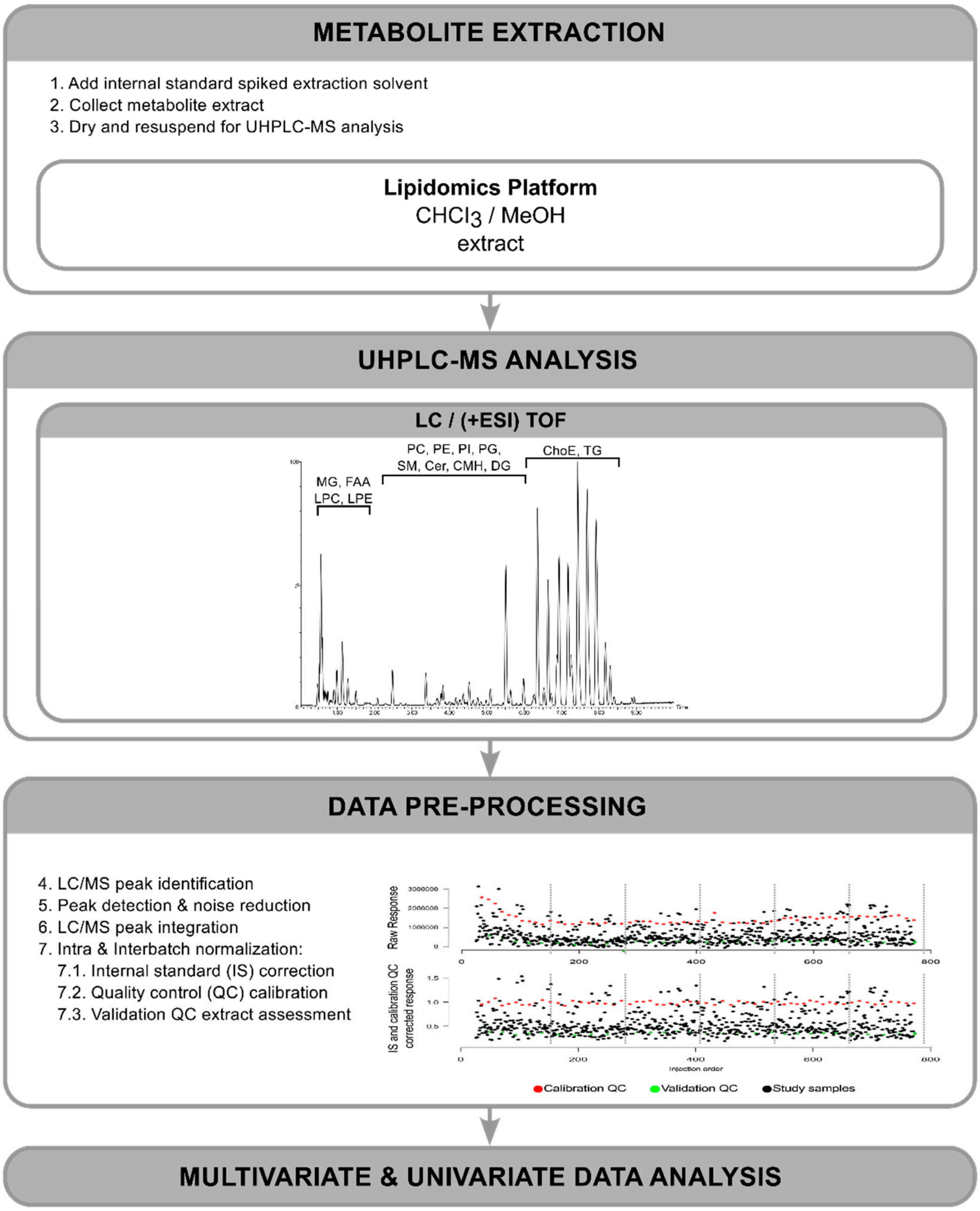


**Figure S1.** Workflow of the UPLC-MS-based targeted metabolomic profiling.

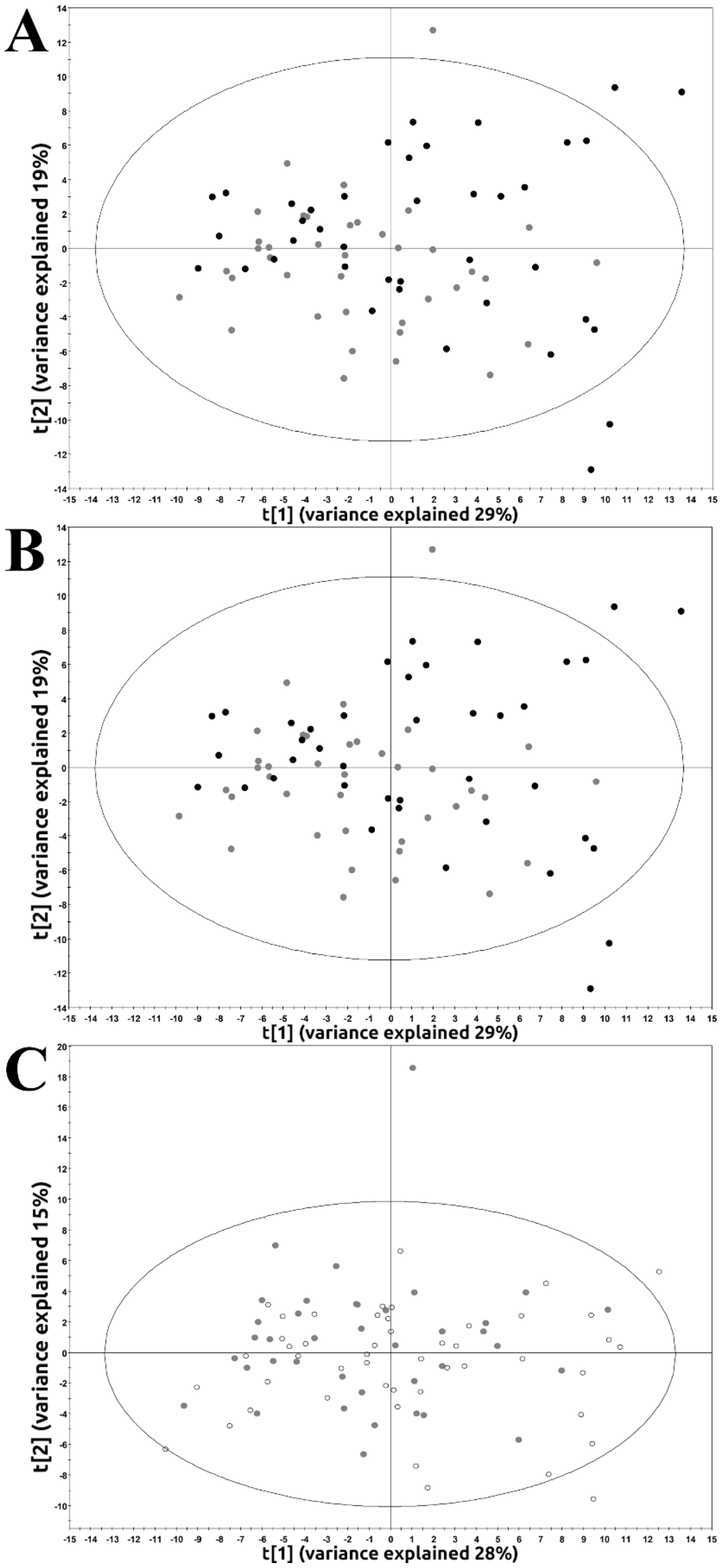


**Figure S2.** Multivariate analysis of paired group. (**A**) CRC vs. AD: R2X = 0.29 and Q2 = 0.24 t[2]: R2X = 0.19 and Q2 = 0.22). Black CRC, grey AD. (**B**) CRC vs. control: R2X = 0.30 and Q2 = 0.25, t[2]: R2X = 0.19 and Q2 = 0.24). Black CRC, white healthy. (**C**) AD vs. control: R2X = 0.28 and Q2 = 0.24, t[2]: R2X = 0.15 and Q2 = 0.15. Grey AD, white healthy.

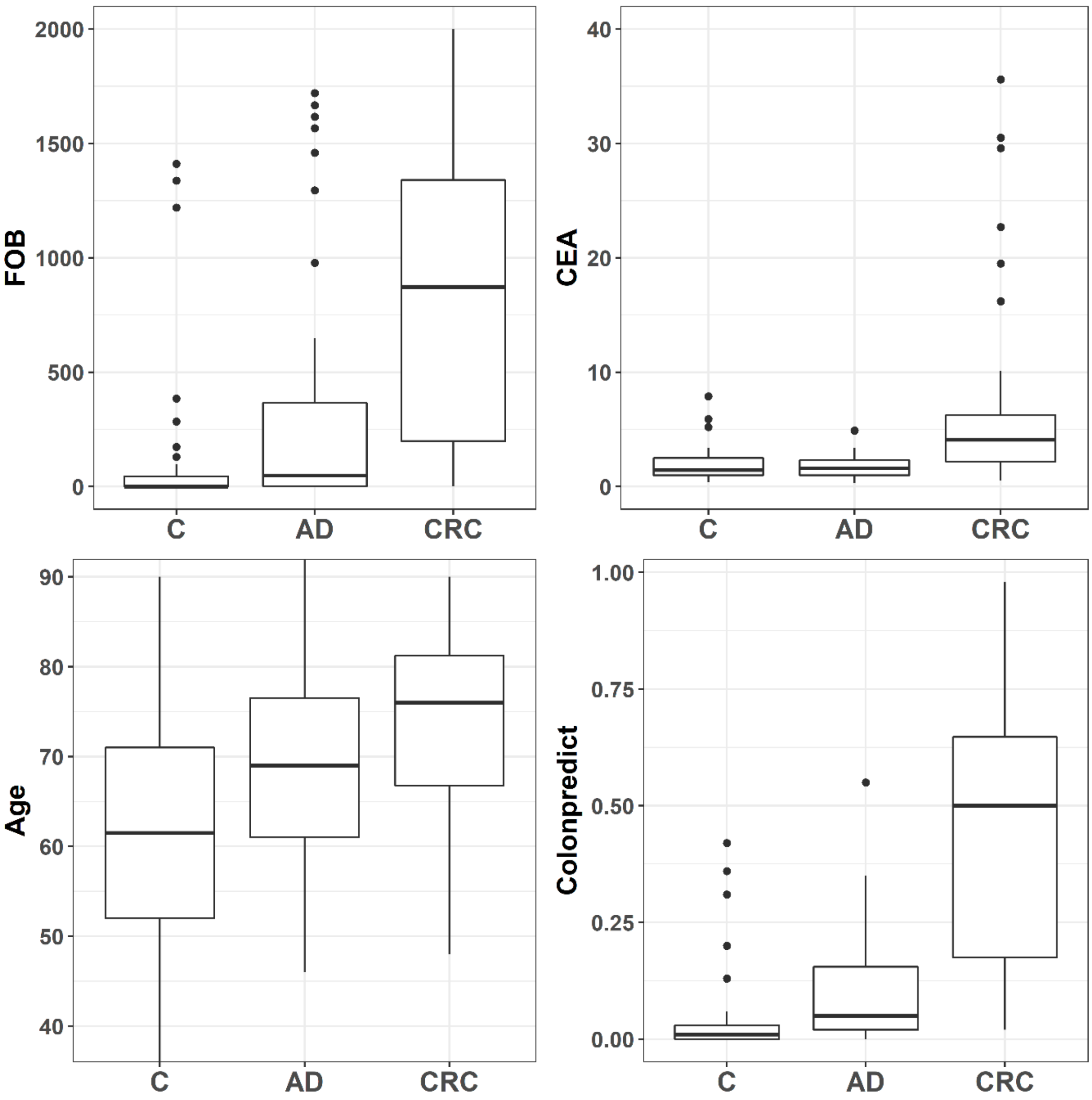


**Figure S3.** Boxplot representation of the clinical parameters distribution on the distinct groups of samples (C, AD and CRC).

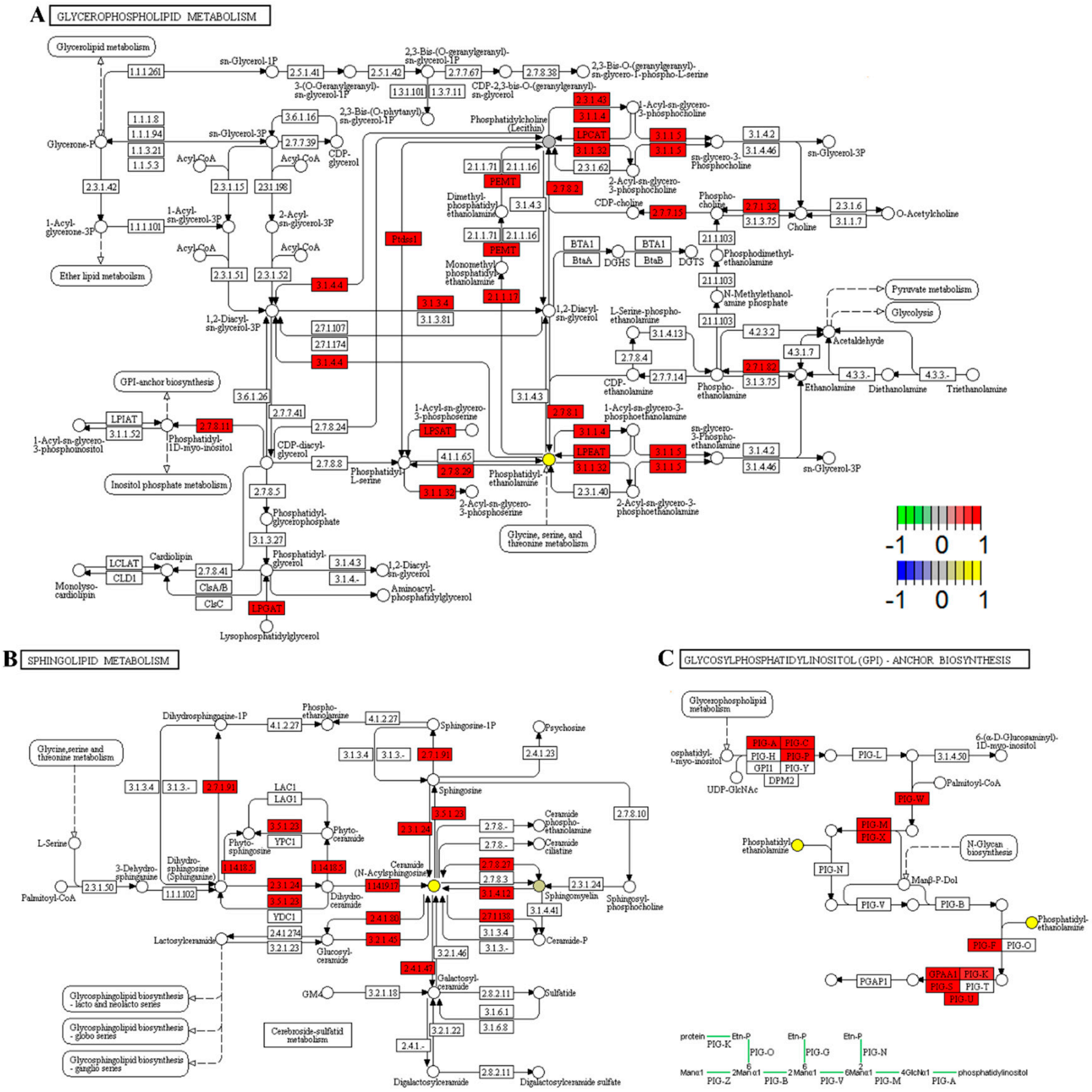


**Figure S4.** Mapping of altered genes and metabolites into the three metabolic pathways identified: sphingolipid metabolism (**A**), glycerophospholipid metabolism (**B**) and glycosylphosphatidylinositol (GPI)-anchor biosynthesis (**C**). Genes detected are coloured in a range green-red, depending on the Fold Change and metabolites in a range blue-yellow.

**Table S2:** Metabolites differentially expressed between control, AD and CRC groups (ANOVA test).

| Family | Metabolite | *p*-Value |
|---|---|---|
| **Cer** | Cer (42:3) | $0.3 \times 10^{-5}$ |
| | Cer (d18:1/16:0) | 0.015 |
| | Cer (d18:1/24:1) + Cer(d18:2/24:0) | 0.008 |
| **ChoE** | ChoE (16:0) | 0.001 |
| | ChoE (18:2) | $0.3 \times 10^{-7}$ |
| | ChoE (20:4) | $0.7 \times 10^{-7}$ |
| **DG** | DG (32:1) | 0.042 |
| **PC** | PC (16:0/16:0) | 0.002 |
| | PC (16:0/18:0) | 0.003 |
| | PC (16:0/18:1) | 0.026 |
| | PC (18:0/18:1) | 0.006 |
| | PC (18:0/20:4) | 0.016 |
| | PC (32:1) | 0.005 |
| | PC (36:3) | 0.028 |
| | PC-O (16:0/16:0) | $0.1 \times 10^{-3}$ |
| | PC-O (34:1) | 0.034 |
| | PC-P (16:0/20:3) | 0.008 |
| **PE** | PE (16:0/18:1) | 0.003 |
| | PE (16:0/18:2) | 0.037 |
| **SM** | SM (42:1) | $0.2 \times 10^{-3}$ |
| | SM (42:3) | $0.1 \times 10^{-5}$ |
| | SM (d18:1/16:0) | $0.8 \times 10^{-4}$ |
| | SM (d18:1/18:0) | 0.027 |
| | SM (d18:1/22:0) | 0.005 |
| | SM (d18:1/23:0) | $0.2 \times 10^{-3}$ |
| | SM (d18:1/24:1) + SM (d18:2/24:0) | $0.4 \times 10^{-5}$ |
| **TG** | TG (49:1) | 0.032 |
| | TG (51:2) | 0.046 |
